# Appropriateness and Associated Factors of Stress Ulcer Prophylaxis for Surgical Inpatients of Orthopedics Department in a Tertiary Hospital: A Cross-Sectional Study

**DOI:** 10.3389/fphar.2022.881063

**Published:** 2022-06-02

**Authors:** Haiyan Li, Ning Li, Xiaoni Jia, Yuyao Zhai, Xiaorong Xue, Yi Qiao

**Affiliations:** ^1^ Department of Pharmacy, Xi’an People’s Hospital (Xi’an Fourth Hospital), The Affiliated Hospital of Northwestern Polytechnical University, Xi’an, China; ^2^ Department of Science and Education, Xi’an Mental Health Center, Xi’an, China; ^3^ Department of Pharmacy, Xi’an Mental Health Center, Xi’an, China; ^4^ Department of Pharmacy, Xijing Hospital, The Fourth Military Medical University, Xi’an, China

**Keywords:** stress ulcer prophylaxis, surgical inpatients, orthopedics department, proton pump inhibitors, clinical pharmacists

## Abstract

**Background:** Stress ulcer prophylaxis (SUP) prescribed in patients admitted to surgical wards with a low risk of stress-related mucosal disease (SRMD) accounted for a considerable proportion of improper use of proton pump inhibitors (PPIs). This study aimed to analyze the appropriateness of SUP prescribing patterns and identify its associated factors in the orthopedics department of a tertiary hospital in the Northwestern China.

**Methods:** In this cross-sectional study, information regarding the demographic and clinical characteristics of 1,200 fracture inpatients who underwent surgical operations from January 2020 to August 2021 were collected from medical records. Established criteria were used to assess the appropriateness of the prescribing pattern for SUP, and the incidence of inappropriate SUP medication was calculated. Logistic regression analyses were used to identify factors associated with inappropriate SUP medication.

**Results:** Approximately, 42.4% of the study population was interpreted as inappropriate prescription of SUP. A total of 397 (33.1%) patients received SUP without a proper indication (overprescription), and the incidence of inappropriate SUP medication was calculated to be 43.11 per 100 patient-days. In addition, 112 (9.3%) inpatients for whom SUP was indicated did not receive SUP (underprescription). PPIs were prescribed in 96.1% of the inpatients who used acid suppression therapy (AST), and intravenous PPIs accounted for 95.3% thereof. In a multivariate logistic regression analysis, age above 65 years and prolonged hospitalization were associated with overprescription of SUP. Increased number of drugs excluding PPIs, the concurrent use of systemic corticosteroids, comorbidity of hypertension, and unemployed or retired status in inpatients were associated with a reduced likelihood of overprescription for SUP. Conversely, prolonged hospitalization, the concurrent use of systemic corticosteroids or anticoagulants, and unemployed status in inpatients were positively associated with underprescription of SUP.

**Conclusion:** There was a high prevalence of inappropriate SUP prescription among noncritically ill inpatients of fracture who underwent surgical operations. We delineated the associated factors with inappropriate SUP medication, which indicated that more information was required for clinicians about rationality and efficiency of their prescribing practices. Effective intervention strategies should be executed by clinical pharmacists to reduce improper SUP medication.

## Introduction

Proton pump inhibitors (PPIs) have become one of the most commonly prescribed medicines, and its consumption continues to increase in recent years worldwide. There is definite evidence that PPIs are being overused in hospitalized patients. Between 25% and 70% of hospitalized patients receive PPIs without an appropriate indication. This means that almost £2 billion worldwide is unnecessarily spent on PPIs every year (Forgacs and Loganayagam, 2008). The inappropriate use of PPIs could not only lead to an increased risk of adverse drug reactions and bodily damage due to the unnecessary use of drugs but also increase the financial burden on patients and healthcare systems. Stress ulceration (SU) is a form of hemorrhagic gastritis, which may occur in patients who have experienced major stressful events in the case of multiple traumas, major surgery, multiple organ failure, heat injury, or sepsis (Anderberg and Sjödahl, 1985). Stress-related mucosal disease (SRMD) is most commonly observed in patients of the intensive care unit (ICU), and prophylaxis against SU should be restricted to such patients while exhibiting a relatively high rate of bleeding and not be routinely recommended in noncritically ill surgical and medical patients (ASHP Therapeutic Guidelines on Stress Ulcer Prophylaxis, 1999). Despite the recommendation, the current status is that more than 22%–88% of hospitalized patients outside the ICU still receive stress ulcer prophylaxis (SUP) without being in risk of developing SRMD or subsequent gastrointestinal bleeding, which represents one of the main reasons for the improper use of PPIs (Heidelbaugh and Inadomi, 2006; Nasser et al., 2010; Issa et al., 2012; Bardou et al., 2015; Savarino et al., 2018). Prior studies have demonstrated that improper SUP medication has been more commonly prescribed in patients admitted to surgical wards, among which orthopedic and general surgeons have prescribed the most PPIs (Mayet, 2007; Craig et al., 2010; Bez et al., 2013; Villamañán et al., 2015; Wijaya et al., 2020).

Inpatients who experience anxiety due to trauma, pain, and starvation during surgical operations are at a higher risk of developing SRMD or subsequent gastrointestinal bleeding. Therefore, SUP is recommended in urgent surgery, complicated procedures, and reoperations (Cook et al., 1994). PPIs could help reduce ulcer-related mortality and the length of hospital stay in elderly patients with femoral neck fractures with a high risk of SU (Singh et al., 2016). However, the perioperative risk of developing gastrointestinal bleeding has been reported to be only roughly 4% (Lalmohamed et al., 2013), while the frequency of nosocomial bleeding occurred in only 0.3% of the patients outside the ICU (Herzig et al., 2011). Considering the extremely low risk of bleeding and the increased risk of adverse events, while lacking direct evidence that SUP medication is beneficial for low-risk patients, the prevention of gastrointestinal bleeding routinely in surgical patients outside the ICU is often inappropriate and unnecessary (ASHP Therapeutic Guidelines on Stress Ulcer Prophylaxis, 1999; Cook and Guyatt, 2018).

So as to promote the rational use of medication and reduce medical costs, the Chinese authorities have taken various measures in recent years. Adjuvant drugs with high prices, larger consumption, and unconfirmed therapeutic effects in their clinical application have been defined as the Key Monitoring Drugs in China. The National Health and Family Planning Commission of the People’s Republic of China (NHFPC) has developed management measures for the Key Monitoring Drugs from 2015. As one of the most commonly prescribed medications, PPIs were included in the list of Key Monitoring Drugs developed by the Health and Family Planning Commission of Anhui, Sichuan, Qinghai, JiangXi, and Shanxi Province from 2015 to 2019. In August 2021, the National Health Commission of the People’s Republic of China indicated that management of PPIs must be scheduled because of abnormally large consumption and the current situation of irrational utilization in hospitals, which meant management targeted at these drugs for improvement had been scheduled. In China, studies have pointed out that SUP without indication account for the majority of inappropriate PPIs prescriptions (Lei, 2017; Luo et al., 2018). Through prescription analysis, we already know from the available publications that inappropriate SUP medication in surgery patients, in particular, patients of the orthopedics department, seems to be more serious (Ma et al., 2018). Between 28.7% and 100.0% of surgical inpatients in the orthopedics department received SUP, but 32.4%–65.8% of inpatients received this therapy without indications (Ruan et al., 2015; Chu et al., 2017; Ma et al., 2018; Yao et al., 2019). To promote the proper prophylactic use of PPIs, the “Consensus Review for SUP and Treatment” was published in 2015 in China and then updated in 2018 (Bo et al., 2018). To further standardize doctors’ prescription behavior of PPIs, the first guideline for the clinical use of PPIs was issued by the National Health Commission of the People’s Republic of China in 2020 (National Health Commission of the People’s Republic of China, 2020).

Although the non-indicated use of acid suppressive medications (ASMs) was commonplace in China, little is known about the SUP prescribing practice for surgery inpatients and its associated factors. Therefore, it is necessary to explore the current situation of SUP prescribing pattern among surgery inpatients, with the goal of providing a basis for future PPIs stewardship in China. We had a testable hypothesis that the prevailing inappropriate prescribing pattern of SUP was high among surgical inpatients of the orthopedics department. The primary objective of this study was therefore to assess the appropriateness of SUP medication for fracture patients who underwent surgical operations in the orthopedics department of a tertiary hospital, including their eligibility, medication choices, and the routes and durations of SUP dosing. The demographic and clinical factors associated with the inappropriate prescription of SUP were clarified for further designing effective interventions to improve the rational utilization of PPIs.

## Materials and Methods

### Study Design and Setting

This cross-sectional study was conducted in a tertiary teaching hospital located in the Shaanxi province of Northwestern China. The hospital had around 1,300 beds in all and 60 beds in the orthopedics department.

### Study Population and Sample Size

The inclusion criteria for participants were inpatients aged ≥12 years who 1) underwent surgical operations in the orthopedics department from January 2020 to August 2021 because of fractures and 2) had a hospital stay length of >3 days. The exclusion criteria were 1) a history of peptic ulcers or gastrointestinal bleeding within 1 year prior to admission; 2) ASMs prescription for the treatment of gastrointestinal diseases such as ulcers, esophagitis, dyspepsia, gastroesophageal reflux disease, or epigastric pain within 1 month prior to admission; 3) new onset of gastrointestinal disease during hospitalization; 4) admission to the ICU or being transferred from or to the ICU halfway; and 5) death during hospitalization.

The minimum number of participants was calculated applying the following formula: *n* = *z* ^2^ *p*(*1-p*)*/d* ^2^, where *n* is the sample size, *z* is the coefficient of confidence interval (1.96), *p* is the prevalence rate, and *d* is the error margin of prevalence (3% *p*). Based on previously published data, SUP for inpatients who underwent surgical operations in the orthopedics department was estimated to be 57.9% (Ruan et al., 2015; Chu et al., 2017; Ma et al., 2018; Yao et al., 2019). As a result, a minimum sample size of 1,041 inpatients was required based on the above assumptions.

**FIGURE 1:**
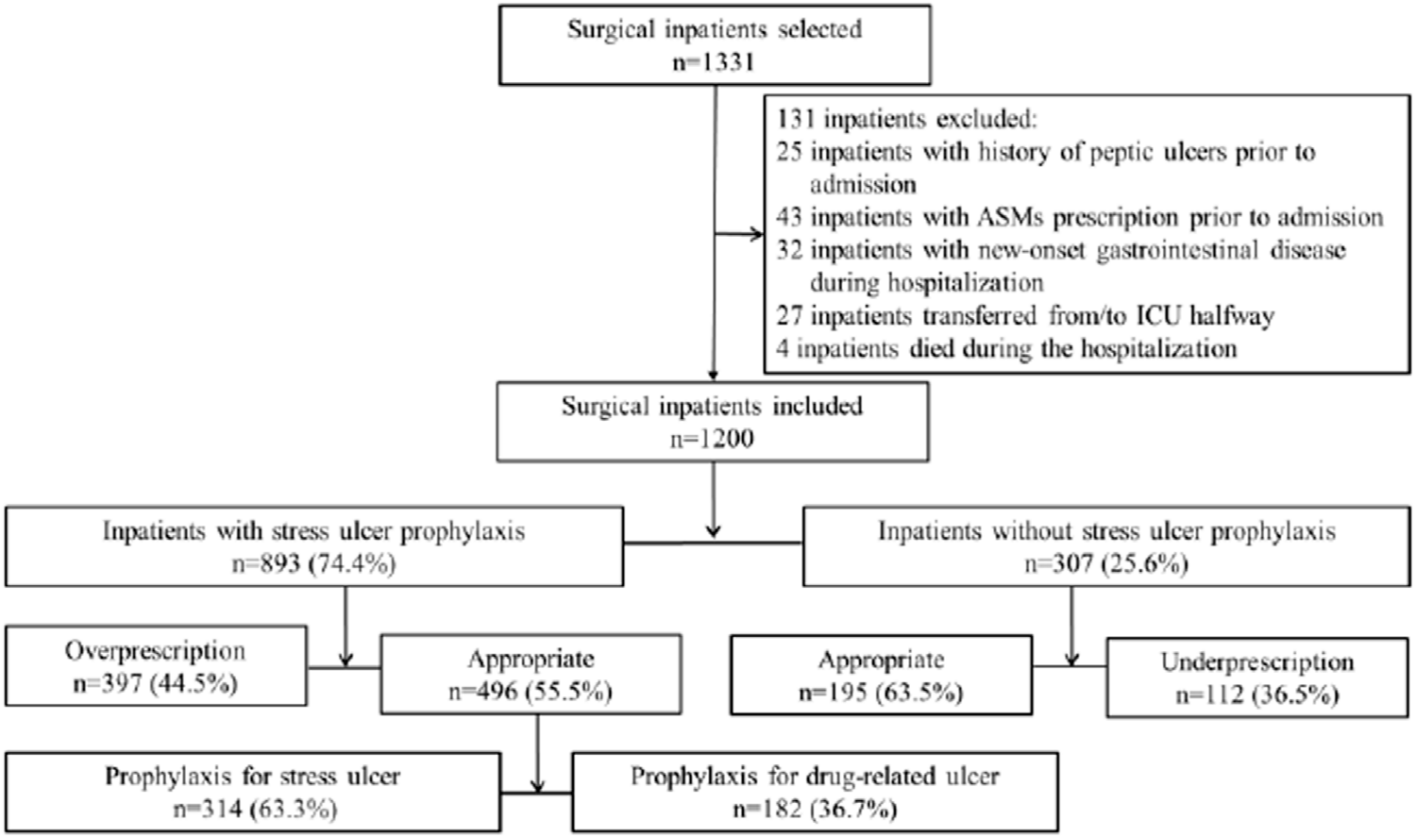
Flow diagram demonstrating inpatient selection and subclassification. The selection and subclassification process for the inpatients who participated in the study is demonstrated. An initial sample size of 1,331 inpatients was selected randomly using a standard computer selection program, and 131 inpatients were excluded according to the criteria determined, and thus 1,200 inpatients were finally recruited in this study. The study inpatients were initially divided into subgroups on the basis of SUP prescription receivers or non–SUP prescription receivers, and then categorized into four groups through ascertaining if the indication was in accordance with the criteria determined. The inpatients who received SUP appropriately were subclassified into two groups according to the criteria determined, of which one met the criteria for SUP indication, while the other met the criteria for drug-induced ulcer prophylaxis.

An initial sample size of 1,331 inpatients who underwent surgical operations because of fracture from January 2020 to August 2021 was selected randomly with a standard computer selection program. A total of 131 inpatients were excluded from the analysis according to the criteria shown above, and thus 1,200 inpatients were finally recruited in this study (Figure 1). Only the first admission was included for patients admitted multiple times during the study period. SUP was defined as the treatment with at least one dose of ASMs initiated in inpatients without any clear indication or any relevant symptom recorded in the medical records. The details of the types of fractures in the patients of our study are provided in the supplementary file (Supplementary Tables S1, S2).

### Criteria Establishment

Based on published evidence-based guidelines and previous literature for the clinical practices of SUP, we established the criteria to evaluate the appropriateness of SUP medication. The claimed SUP indication group was subclassified as 1) meeting the criteria for SUP indication or 2) meeting the criteria for drug-induced ulcer prophylaxis (Figure 1). SUP medication was judged to be appropriate if the surgical inpatient had one major or at least two minor risk factors (Cook et al., 1994; ASHP Therapeutic Guidelines on Stress Ulcer Prophylaxis, 1999; Bez et al., 2013; Bo et al., 2018; National Health Commission of the People’s Republic of China, 2020) (Table 1). Since nonsteroidal anti-inflammatory drugs (NSAIDs) were required for pain management, anticoagulants for deep vein thrombosis prophylaxis and the concomitant use of ulcerogenic medicine for comorbidities and acknowledged risk factors for drug-related ulcer prophylaxis were also established (García Rodríguez and Jick, 1994; Bhatt et al., 2008; Lanza et al., 2009; Bez et al., 2013) (Table 2). The prescription of ASMs was considered appropriate if the above risk factors were present.

**TABLE 1:** Risk factors for stress ulcer (Cook et al., 1994; ASHP Therapeutic Guidelines on Stress Ulcer Prophylaxis, 1999; Bez et al., 2013; Bo et al., 2018; National Health Commission of the People’s Republic of China, 2020).

**The presence of one major risk factor from the following:**
1 Respiratory failure: mechanical ventilation >48 h
2 Coagulopathy: platelet count <50,000/mm^3^ (50 × 10^9^/L), international normalized ratio >1.5, or partial thromboplastin time >2.0 times the control value
3 Experiencing surgical operation for more than 3 h
4 Head injury with a Glasgow Coma Score of ≤10 or an inability to obey simple commands
5 Thermal injury involving >35% of the body surface area
6 Partial hepatectomy
7 Hepatic or renal transplantation
8 Multiple traumas with the Injury Severity Score of ≥16
9 Acute renal failure or hepatic failure
10 Traumatic brain injury or spinal cord injury
**The presence of at least two minor risk factors of the following:**
1 Sepsis
2 Occult or overt bleeding for ≥6 days
3 Corticosteroid therapy (>250 mg/d hydrocortisone or equivalent daily)

**TABLE 2:** Risk factors for drug-related ulcer (García Rodríguez and Jick, 1994; Bhatt et al., 2008; Lanza et al., 2009; Bez et al., 2013).

**Risk factor**
1 High-dose NSAID therapy (ibuprofen >1,500 mg daily, diclofenac >100 mg daily, or mefenamic acid >1,250 mg daily)
2 Concomitant NSAID use with antiplatelet agents (including low-dose aspirin), corticosteroids, or anticoagulants
3 Age >65 years, concomitant use of NSAID
4 Concomitant anticoagulant use with antiplatelet agents
5 Dual antiplatelet therapies
6 Age ≥60 years, concomitant corticosteroid use with antiplatelet agents

### Data Collection

The sociodemographic and medical variables were collected from the hospital information system (HIS) by reviewing the electronic medical records. Sociodemographic information of inpatients included data on age (years), gender (male or female), current smokers (yes or no), alcohol consumption (yes or no), occupational status (employed, unemployed, or retired), place of residence (urban or rural), and health insurance (insured or uninsured). The data extracted from the medical records included the following variables: diagnosis at admission, comorbidity conditions (hypertension, diabetes mellitus, coronary artery disease, and osteoporosis), total number of comorbidities, complications (limb vein thrombosis, respiratory infection, urinary infection, and bedsore), admission/discharge date, length of hospital stay (days), name and duration of surgical operations, pertinent laboratory data, number of drugs excluding PPIs during hospitalization, and adverse drug reactions during hospitalization. ASMs prescription for inpatients included information on generic names, drug specifications, units, total doses, manufacturers, ASMs concerning routes, and frequencies and durations of administrations. Co-medications potentially influencing the prescription pattern of ASMs were also reviewed to identify the associated factors of SUP, including antiplatelet agents (aspirin, clopidogrel, and prasugrel), anticoagulants (warfarin, low-molecular heparin, rivaroxaban, and apixaban), systemic corticosteroids (hydrocortisone, dexamethasone, and methylprednisolone), and NSAIDs (celecoxib, flurbiprofen, ibuprofen, aceclofenac, parecoxib, loxoprofen, ketochromate tromethamine, and diclofenac). Five branded and generic PPIs were available as both oral and intravenous preparations in our hospital when this study was conducted. The defined daily dose (DDD) of PPIs taken by the participants was identified according to the Anatomical and Therapeutic Classification (ATC) code A02BC (Table 3).

**TABLE 3:** Proton pump inhibitors available in our hospital.

Drug	DDD (mg)	ATC code
Omeprazole	20	A02BC01
Rabeprazole	20	A02BC04
Lansoprazole	30	A02BC03
Esomeprazole	30	A02BC05
Pantoprazole	40	A02BC02

### Outcome Measurements

Our primary outcome variable was the appropriateness evaluation of SUP prescribing patterns for fracture patients who underwent surgical operations in the orthopedics department. Factors influencing inappropriate prescription of SUP were analyzed as another end point in our study.

### Statistical Analysis

The collected data were analyzed to assess the appropriateness of ASMs prescription and identify the associated factors of inappropriate SUP medication. The appropriateness of SUP medication was determined according to the criteria listed above. Patients were initially divided into subgroups on the basis of SUP prescription receivers or non–SUP prescription receivers and were then categorized into four groups through ascertaining if the indication was in accordance with the abovementioned criteria: those who received SUP appropriately, those who received SUP without a proper indication (overprescription group), those who were appropriate non–SUP prescription receivers, and those for whom SUP was indicated for but did not receive SUP (underprescription group). Inappropriate dosage of ASMs was noted but was not included in the over- or underprescription groups. The defined daily doses per 100 patient-days (DDDs/100 PDs) were used for measuring the consumption of PPIs for SUP. ASMs prescription of the overprescription group were counted as inappropriate. Based on a previous study (Masood et al., 2018), incidence of inappropriate SUP medication was evaluated, which was calculated as the inappropriate patient-days divided by total patient-days receiving prescription of ASMs and then converted to incidence per 100 patient-days. To identify factors associated with inappropriate SUP medication, patients receiving overprescription and appropriate prescription of SUP were compared. Similarly, patients receiving underprescription were compared with non–SUP prescription receivers.

The skew continuous variables of demographic and clinical data are presented as median (interquartile range) after normality test. Categorical variables are presented as frequency (percentages). Differences in demographic and clinical data were evaluated using the Mann–Whitney test or Pearson chi-squared test as appropriate. After the univariate models were estimated for each predictive variable, the multivariate logistic regression models were used to investigate independent factors associated with the inappropriate prescription of SUP. All analyses were performed using the SPSS V25.0 Statistical Software Package for Windows. The level of statistical significance was set to 0.05.

## Results

### Characteristics of Study Participants

A total of 893 inpatients with ASMs prescription for SUP had a median age of 45 (58, 70) years and the majority (50.3%) were female; 307 inpatients without ASMs prescription for SUP had a median age of 49 (33, 62) years and the majority (59.9%) were male.

### Appropriateness of Stress Ulcer Prophylaxis Prescribing Patterns

SUP medication was prescribed to 893 inpatients (74.4%) of the study population, and the consumption of PPIs was 57.90 DDDs per 100 patient-days (PDs). A total of 509 inpatients (42.4%) of the study population were interpreted as having received an inappropriate prescription of SUP, of which 397 (33.1%) and 112 (9.3%) were interpreted as having received overprescription and underprescription of SUP, respectively. The incidence of inappropriate SUP medication was calculated to be 43.11 per 100 patient-days. PPIs were prescribed to 96.1% of the inpatients using acid suppression therapy, in whom intravenous PPIs accounted for 95.3%. Pantoprazole was prescribed in 46.7% of the inpatients who were prescribed PPIs, followed by omeprazole (29.7%), lansoprazole (13.2%), and esomeprazole (10.4%). Rabeprazole was not prescribed. Cimetidine injection was the only histamine-2-receptor antagonist (H_2_RAs) prescribed in our study. Only 35 inpatients received cimetidine injection, while 17 inpatients received PPIs injection followed or preceded by cimetidine injection sequentially. The mean duration of SUP medication was 3.65 ± 3.24 days a total of 496 inpatients (41.3%) were judged to meet the criteria for appropriate SUP, of whom 182 (36.7%) received prophylaxis against drug-related ulcer and 314 (63.3%) received prophylaxis against SU. Patients who received surgical operation for more than 3 h accounted for almost two-thirds of the 314 inpatients with appropriate indications for prophylaxis against SU (Figure 1). ASMs prescription patterns for SUP are shown in Table 4.

**TABLE 4:** ASMs prescription patterns for SUP.

Appropriate prescription for SUP	Routes of administration		Duration of administration (days)	DDDs/100 PDs of PPIs
	Intravenous	Oral		
Yes	490	6	3.74 ± 3.13	55.93
No^a^	396	1	3.54 ± 3.37	60.23
Total	886	7	3.65 ± 3.24	57.90

a This indicates the overprescription group.

### Associated Factors of Inappropriate Stress Ulcer Prophylaxis Medication

Demographic and clinical variables of inpatients with ASMs prescription for SUP are shown in Table 5 and those of inpatients without ASMs prescription for SUP are shown in Table 6.

**TABLE 5:** Demographic and clinical characteristics of inpatients with ASMs prescription for SUP.

Characteristics	Total (*n* = 893)	Appropriate (*n* = 496)	Overprescription (*n* = 397)	*p*-value
Age (years)				0.001
Median (Q_1_, Q_3_)	45 (58, 70)	56 (45, 67.5)	61 (46, 73)	
≤44	213 (23.8)	122 (24.6)	91 (22.9)	<0.001
45–64	357 (40.0)	223 (45.0)	134 (33.8)	
≥65	323 (36.2)	151 (30.4)	172 (43.3)	
Gender				0.725
Female	449 (50.3)	252 (50.8)	197 (49.6)	
Male	444 (49.7)	244 (49.2)	200 (50.4)	
Current smokers				0.342
No	737 (82.5)	404 (81.5)	333 (83.9)	
Yes	156 (17.5)	92 (18.5)	64 (16.1)	
Alcohol consumption				0.039
No	790 (88.5)	429 (86.5)	361 (90.9)	
Yes	103 (11.5)	67 (13.5)	36 (9.1)	
Occupational status				<0.001
Employed	255 (28.6)	105 (21.2)	150 (37.8)	
Unemployed	453 (50.7)	286 (57.6)	167 (42.1)	
Retired	185 (20.7)	105 (21.2)	80 (20.1)	
Residence				0.009
Rural	473 (53.0)	282 (56.9)	191 (48.1)	
Urban	420 (47.0)	214 (43.1)	206 (51.9)	
Health insurance				0.143
No	303 (33.9)	158 (31.9)	145 (36.5)	
Yes	590 (66.1)	338 (68.1)	252 (63.5)	
Comorbidity conditions				
Hypertension	225 (25.2)	128 (25.8)	97 (24.4)	0.639
Diabetes mellitus	111 (12.4)	58 (11.7)	53 (13.4)	0.456
Coronary artery disease	130 (14.6)	55 (11.1)	75 (18.9)	0.001
Osteoporosis	76 (8.5)	44 (8.9)	32 (8.1)	0.666
Number of comorbidities	0 (0, 1)	0 (0, 1)	1 (0, 2)	0.003
Complications	127 (14.2)	53 (10.7)	74 (18.6)	0.001
Concurrently used drugs				
Anticoagulants	531 (59.5)	279 (56.3)	252 (63.5)	0.029
Antiplatelet agents	24 (2.7)	16 (3.2)	8 (2.0)	0.266
Systemic corticosteroids	345 (38.6)	258 (52.0)	87 (21.9)	<0.001
NSAIDs	722 (80.9)	389 (78.4)	333 (83.9)	0.040
Number of drugs excluding PPIs				<0.001
Median (Q_1_, Q_3_)	19 (14, 28)	21 (15.5, 29)	16 (12, 25)	
6–14	263 (29.5)	102 (20.6)	161 (40.6)	<0.001
15–19	212 (23.7)	116 (23.4)	96 (24.2)	
20–28	217 (24.3)	141 (28.4)	76 (19.1)	
29–59	201 (22.5)	137 (27.6)	64 (16.1)	
Length of hospital stay (days)				0.172
Median (Q_1_, Q_3_)	10 (7, 16)	11 (8, 15)	10 (7, 16)	
3–7	230 (25.8)	111 (22.4)	119 (30.0)	0.011
8–10	224 (25.1)	131 (26.4)	93 (23.4)	
11–16	244 (27.3)	152 (30.6)	92 (23.2)	
17–54	195 (21.8)	102 (20.6)	93 (23.4)	

The data are presented as numbers (proportions) or the median (interquartile range). Bold values indicate a *p*-value <0.05.

**TABLE 6:** Demographic and clinical characteristics of the inpatients without ASMs prescription for SUP.

Characteristics	Total (*n* = 307)	Appropriate (*n* = 195)	Underprescription (*n* = 112)	*p-value*
Age (years)				0.066
Median (Q_1_, Q_3_)	49 (33, 62)	46 (31, 61)	51.5 (36, 62)	
≤44	133 (43.3)	90 (46.1)	43 (38.4)	0.375
45–64	115 (37.5)	68 (34.9)	47 (42.0)	
≥65	59 (19.2)	37 (19.0)	22 (19.6)	
Gender				0.057
Female	123 (40.1)	86 (44.1)	37 (33.0)	
Male	184 (59.9)	109 (55.9)	75 (67.0)	
Current smokers				0.112
No	250 (81.4)	164 (84.1)	86 (76.8)	
Yes	57 (18.6)	31 (15.9)	26 (23.2)	
Alcohol consumption				0.596
No	273 (88.9)	172 (88.2)	101 (90.2)	
Yes	34 (11.1)	23 (11.8)	11 (9.8)	
Occupational status				<0.001
Employed	126 (41.0)	97 (49.7)	29 (25.9)	
Unemployed	126 (41.0)	59 (30.3)	67 (59.8)	
Retired	55 (17.9)	39 (20.0)	16 (14.3)	
Residence				<0.001
Rural	136 (44.3)	71 (36.4)	65 (58.0)	
Urban	171 (55.7)	124 (63.6)	47 (42.0)	
Health insurance				0.461
No	104 (33.9)	69 (35.4)	35 (31.2)	
Yes	203 (66.1)	126 (64.6)	77 (68.8)	
Comorbidity conditions				
Hypertension	50 (16.3)	28 (14.4)	22 (19.6)	0.227
Diabetes mellitus	18 (5.9)	12 (6.2)	6 (5.4)	0.775
Coronary artery disease	15 (4.9)	9 (4.6)	6 (5.4)	0.772
Osteoporosis	21 (6.8)	12 (6.2)	9 (8.0)	0.529
Number of comorbidities	0 (0, 1)	0 (0, 0)	0 (0, 1)	0.370
Complications	19 (6.2)	8 (4.1)	11 (9.8)	0.045
Concurrently used drugs				
Anticoagulants	100 (32.6)	44 (22.6)	56 (50)	<0.001
Systemic corticosteroids	46 (15.0)	16 (8.2)	30 (26.8)	<0.001
NSAIDs	203 (66.1)	125 (64.1)	78 (69.6)	0.323
Number of drugs excluding PPIs				0.028
Median (Q_1_, Q_3_)	14 (10, 19)	13 (10, 18)	15 (12, 20.5)	
3–10	79 (25.7)	60 (30.8)	19 (17.0)	0.064
11–14	86 (28.0)	51 (26.2)	35 (31.3)	
15–19	69 (22.5)	42 (21.5)	27 (24.1)	
20–57	73 (23.8)	42 (21.5)	31 (27.7)	
Length of hospital stay (days)				<0.001
Median (Q_1,_ Q_3_)	10 (6, 15)	8 (6, 12)	12 (8, 19)	
3–6	81 (26.4)	64 (32.8)	17 (15.2)	<0.001
7–10	94 (30.6)	68 (34.9)	26 (23.2)	
11–15	67 (21.8)	40 (20.5)	27 (24.1)	
16–65	65 (21.2)	23 (11.8)	42 (37.5)	

The data are presented as numbers (proportions) or the median (interquartile range). Bold values indicate a *p*-value <0.05.

When compared with inpatients who received SUP appropriately, nine factors were significantly associated with overprescription of ASMs for SUP practice (*p* < 0.05): age above 65 years, alcohol consumption, unemployed status, living in urban areas, comorbidity of coronary artery disease, complications, the concurrent use of anticoagulants, systemic corticosteroids or NSAIDs, number of drugs excluding PPIs, and length of hospital stay (Table 7). When compared with appropriate non-SUP prescription, five factors were significantly associated with underprescription of ASMs for SUP (*p* < 0.05): unemployed status, living in urban areas, the concurrent use of anticoagulants or systemic corticosteroids, number of drugs excluding PPIs, and length of hospital stay (Table 8).

**TABLE 7:** Univariate and multivariate logistic regression analyses of factors associated with overprescription of ASMs for SUP.

Characteristics	Unadjusted OR (95% CI)	*p-value*	Adjusted OR (95% CI)	*p-value*
Age (years)				
≤44	1.000 (Reference)		1.000 (Reference)	
45–64	0.806 (0.570–1.138)	0.221	1.193 (0.797–1.786)	0.391
≥65	1.527 (1.078–2.164)	0.017	3.591 (2.145–6.012)	<0.001
Gender				
Female	1.000 (Reference)		1.000 (Reference)	
Male	1.049 (0.805–1.365)	0.725	1.200 (0.851–1.693)	0.299
Current smokers				
No	1.000 (Reference)		1.000 (Reference)	
Yes	0.844 (0.594–1.198)	0.343	1.076 (0.640–1.809)	0.782
Alcohol consumption				
No	1.000 (Reference)		1.000 (Reference)	
Yes	0.639 (0.416–0.980)	**0.040**	0.638 (0.350–1.163)	0.142
Occupational status				
Employed	1.000 (Reference)		1.000 (Reference)	
Unemployed	0.409 (0.299–0.560)	**<0.001**	0.457 (0.293–0.713)	**0.001**
Retired	0.533 (0.364–0.782)	0.001	0.287 (0.171–0.480)	<0.001
Residence				
Rural	1.000 (Reference)		1.000 (Reference)	
Urban	1.421 (1.090–1.853)	**0.009**	1.204 (0.791–1.833)	0.386
Health insurance				
No	1.000 (Reference)		1.000 (Reference)	
Yes	0.812 (0.615–1.073)	0.143	0.779 (0.559–1.085)	0.140
Comorbidity conditions				
Hypertension	0.930 (0.685–1.261)	0.639	0.663 (0.452–0.972)	0.035
Diabetes mellitus	1.163 (0.781–1.733)	0.456	1.035 (0.645–1.663)	0.886
Coronary artery disease	1.868 (1.282–2.721)	**0.001**	1.485 (0.934–2.359)	0.095
Osteoporosis	0.901 (0.560–1.449)	0.666	0.877 (0.511–1.506)	0.634
Number of comorbidities	1.108 (0.947–1.296)	0.199	-	
Complication				
No	1.000 (Reference)		1.000 (Reference)	
Yes	1.915 (1.309–2.802)	**0.001**	1.528 (0.984–2.372)	0.059
Concurrently used drugs				
Anticoagulants	1.352 (1.031–1.772)	**0.029**	1.008 (0.719–1.413)	0.964
Antiplatelet agents	0.617 (0.261–1.457)	0.271	0.542 (0.204–1.443)	0.220
Systemic corticosteroids	0.259 (0.193–0.348)	**<0.001**	0.316 (0.224–0.446)	**<0.001**
NSAIDs	1.431 (1.016–2.016)	0.040	1.432 (0.964–2.125)	0.075
Number of drugs excluding PPIs				
6–14	1.000 (Reference)		1.000 (Reference)	
15–19	0.524 (0.363–0.757)	**0.001**	0.602 (0.397–0.911)	**0.016**
20–28	0.341 (0.235–0.496)	**<0.001**	0.415 (0.263–0.656)	**<0.001**
29–59	0.296 (0.201–0.436)	**<0.001**	0.348 (0.205–0.590)	**<0.001**
Length of hospital stay (days)				
3–7	1.000 (Reference)		1.000 (Reference)	
8–10	0.662 (0.457–0.959)	**0.029**	0.860 (0.561–1.317)	0.487
11–16	0.565 (0.392–0.814)	**0.002**	1.209 (0.772–1.892)	0.407
17–54	0.850 (0.580–1.256)	0.406	1.838 (1.122–3.009)	**0.016**

Bold values indicate a *p*-value <0.05. OR, odds ratio; CI, confidence interval.

**TABLE 8:** Univariate and multivariate logistic regression analysis of factors associated with underprescription of ASMs for SUP.

Characteristic	Unadjusted OR (95% CI)	*p-value*	Adjusted OR (95% CI)	*p-value*
Age (years)				
≤44	1.000 (Reference)		1.000 (Reference)	
45–64	1.447 (0.860–2.433)	0.164	1.037 (0.522–2.058)	0.918
≥65	1.245 (0.656–2.362)	0.503	1.315 (0.463–3.737)	0.607
Gender				
Female	1.000 (Reference)		1.000 (Reference)	
Male	1.599 (0.985–2.597)	0.058	1.916 (0.963–3.814)	0.064
Current smokers				
No	1.000 (Reference)		1.000 (Reference)	
Yes	1.599 (0.893–2.865)	0.114	2.272 (0.919–5.616)	0.076
Alcohol consumption				
No	1.000 (Reference)		1.000 (Reference)	
Yes	0.814 (0.381–1.740)	0.596	0.558 (0.182–1.711)	0.308
Occupational status				
Employed	1.000 (Reference)		1.000 (Reference)	
Unemployed	3.798 (2.208–6.536)	**<0.001**	2.397 (1.075–5.346)	**0.033**
Retired	1.372 (0.672–2.804)	0.385	1.098 (0.356–3.390)	0.871
Residence				
Rural	1.000 (Reference)		1.000 (Reference)	
Urban	0.414 (0.257–0.666)	**<0.001**	0.813 (0.379–1.743)	0.595
Health insurance				
No	1.000 (Reference)		1.000 (Reference)	
Yes	1.205 (0.734–1.978)	0.461	1.059 (0.552–2.028)	0.864
Comorbidity conditions				
Hypertension	1.458 (0.789–2.695)	0.229	2.075 (0.929–4.636)	0.075
Diabetes mellitus	0.863 (0.315–2.367)	0.775	0.323 (0.082–1.268)	0.105
Coronary artery disease	1.170 (0.405–3.377)	0.772	1.103 (0.278–4.375)	0.890
Osteoporosis	1.333 (0.543–3.269)	0.531	1.220 (0.412–3.608)	0.719
Number of comorbidities	1.183 (0.830–1.687)	0.352	—	
Complication				
No	1.000 (Reference)		1.000 (Reference)	
Yes	2.546 (0.992–6.532)	0.052	1.035 (0.326–3.285)	0.954
Concurrently used drugs				
Anticoagulants	3.432 (2.082–5.658)	**<0.001**	2.427 (1.257–4.684)	**0.008**
Systemic corticosteroids	4.093 (2.114–7.924)	**<0.001**	4.548 (1.988–10.405)	**<0.001**
NSAIDs	1.285 (0.781–2.114)	0.324	1.159 (0.632–2.126)	0.632
Number of drugs excluding PPIs				
3–10	1.000 (Reference)		1.000 (Reference)	
11–14	2.167 (1.107–4.243)	**0.024**	1.346 (0.610–2.970)	0.462
15–19	2.030 (1.001–4.117)	0.050	0.830 (0.337–2.044)	0.685
20–57	2.331 (1.164–4.665)	0.017	0.394 (0.144–1.081)	0.071
Length of hospital stay (days)				
3–6	1.000 (Reference)		1.000 (Reference)	
7–10	1.439 (0.715–2.899)	0.308	1.543 (0.684–3.478)	0.296
11–15	2.541 (1.232–5.243)	**0.012**	2.029 (0.849–4.848)	0.112
16–65	6.875 (3.287–14.378)	**<0.001**	5.935 (2.302–15.300)	**<0.001**

Bold values indicate a *p*-value < 0.05. OR, odds ratio; CI, confidence interval.

In multivariate logistic regression analysis, age above 65 years and prolonged hospitalization were associated with overprescription of SUP. Increased number of drugs excluding PPIs, the concurrent use of systemic corticosteroids, comorbidity of hypertension, and unemployed or retired status in inpatients were associated with reduced likelihood of overprescription for SUP (Table 7). Conversely, prolonged hospitalization, the concurrent use of systemic corticosteroids or anticoagulants, and unemployed status in inpatients were positively associated with underprescription of SUP (Table 8).

There were nine cases of reversible disturbances such as nausea, headache, diarrhea, abdominal pain, constipation, flatulence, dizziness, and anaphylactic reactions documented during the study period.

## Discussion

Our study highlights the prevalence of inappropriate SUP medication in noncritically ill fracture patients who underwent surgical operations. Approximately, 42.4% of the study population were interpreted as inappropriate prescription of SUP. The literature had revealed that 48%, 61.6%, and 69% of inpatients in the surgery department were found to be inappropriately prescribed PPIs for SUP (Nasser et al., 2010; Bez et al., 2013; Wijaya et al., 2020), which is higher than the data observed in our study. Our study indicates that approximately 33.1% of fracture patients who underwent surgical operations might not require intravenous PPIs for SUP on a routine basis, which is lower than published observations in China (Ma et al., 2018; Yao et al., 2019). The incidence of inappropriate SUP medication was calculated to be 43.11 per 100 patient-days, which is higher than 26.75 per 100 patient-days in an academic medical ICU (Masood et al., 2018). A previous study has revealed that 33% of patients who were although SUP candidates did not receive ASMs (Issa et al., 2012), which is higher than 9.3% in our study. The total consumption of PPIs in our study was 60.23 DDDs/100 PDs. Therefore, efforts to reduce improper SUP medication are urgently and crucially required.

Based on recent published studies, PPIs seem to be more effective than H_2_RAs for SUP (Bardou et al., 2015). PPIs were more extensively prescribed for the prophylaxis and therapy of NSAID- and aspirin-associated gastrointestinal bleeding (Bhatt et al., 2008; Lanza et al., 2009). In our study, 96.1% of the patients were prescribed PPIs, which is consistent with the current practice trends (Bo et al., 2018; Issa et al., 2012; National Health Commission of the People’s Republic of China, 2020). The Food and Drug Administration has currently approved omeprazole as the only PPI for SUP in critically ill patients (Bez et al., 2013). One study indicated that lansoprazole was not recommended for SUP (Allen et al., 2004). Pantoprazole, followed by omeprazole, was the most commonly prescribed PPIs in our study. A possible explanation could be that pantoprazole was included in various surgical procedures in hospitals (Villamañán et al., 2015), and pantoprazole might be preferred in clopidogrel users for lacking inhibition of hepatic CYP 2C19 (Savarino et al., 2018). In addition, as the only PPI listed among national essential medicines of China, omeprazole should be preferred according to policy guidance of the authorities in China. PPIs twice daily (omeprazole 20 mg, rabeprazole 20 mg, lansoprazole 30 mg, esomeprazole 30 mg, and pantoprazole 40 mg) were recommended for prophylaxis against stress ulcer according to the Chinese guidelines (Bo et al., 2018; National Health Commission of the People’s Republic of China, 2020). Unfortunately, there is a lack of recommendation or consensus on PPI dose for prophylaxis against drug-related ulcer in the current literature.

Our study demonstrated the prevalence of intravenous PPIs for SUP, which occurred in approximately 95.3% of the inpatients using ASMs. Based on the published study, injections given to inpatients who have nil-by-mouth conditions or experience severe motility disorders have been considered appropriate (Wijaya et al., 2020). All the inpatients were admitted to the orthopedics ward outside the ICU in our study, most of whom could receive food intake by mouth and could be given oral therapy. In our study, 62% of the inpatients had inappropriate routes of administration including unnecessary intravenous administration when oral formulations would be more appropriate. Prior published studies have demonstrated that inappropriate routes of drug administration account for 42.7% or 45% of the preparations used (Nasser et al., 2010; Luo et al., 2018), which is lower than the observations made in our study. Another study demonstrated that the incidence of omeprazole being administered *via* inaccurate routes was 96.7% (Wijaya et al., 2020), which is higher than that observed in our study. The effectiveness of oral PPIs was similar to injectable formulations with equivalent doses but with cheaper prices and fewer complications related to intravenous administration (Nasser et al., 2010; Wijaya et al., 2020). This highlights the need for clinical pharmacists to intervene and suggest appropriate routes of drug administration for inpatients.

SUP should be started at the onset of risk factors and continued beyond the high-risk period (Allen et al., 2004; Bo et al., 2018; National Health Commission of the People’s Republic of China, 2020), while prophylaxis should be discontinued when risk factors have been resolved (ASHP Therapeutic Guidelines on Stress Ulcer Prophylaxis, 1999). In our study, the mean duration of SUP was 3.65 ± 3.24 days; 246 inpatients (27.6%) had received ASMs for more than 5 days and 3 of them for up to more than 20 days. Based on the literature review, most patients received intravenous PPIs for claimed SUP indication for approximately 5 days or a mean duration of 6.3 ± 4.5 (SD) days, respectively (Lam et al., 1999; Nasser et al., 2010), which appeared to be longer than the findings in our study. This might be explained by the fact that physicians did not reassess the need for PPIs use regularly (Bez et al., 2013). In addition, due to heavy work and misunderstanding “longer duration for better effect,” surgeons have always ignored or prolonged the duration of prophylaxis (Luo et al., 2018).

Age above 65 years was a predictor of overprescription for SUP in our study. This was supported by published data which states that older age was a significant variable predicting inappropriate AST use (Afif et al., 2007; Issa et al., 2012). In a large cohort of noncritically ill hospitalized patients, age >60 years was identified as an independent risk factor for nosocomial gastrointestinal bleeding (Herzig et al., 2013). Furthermore, comorbidities did increase with age, and older patients were often potentially precarious during hospitalization (Afif et al., 2007), as a result of which the use of ASMs was understandable. Nonetheless, the use of ASMs must be individualized.

Our study shows that increased number of drugs excluding PPIs was associated with a decreased risk of overprescription of ASMs for SUP, which suggests that clinicians are more cautious about prescribing ASMs for patients on multiple drug treatments. The findings in our study are inconsistent with the current literature. One study had indicated that the only independent predictor of inappropriate PPIs use was the number of medications (Voukelatou et al., 2019). Another analysis also indicated that the total number of drugs excluding PPIs was the predictor of overprescribed PPIs (Jarchow-Macdonald and Mangoni, 2013). Our study also shows that comorbidity of hypertension is associated with a decreased risk of overprescription of ASMs for SUP, which indicates that clinicians have been more cautious about prescribing ASMs for hypertension patients. According to the literature, cardiology patients were often maintained on aspirin and other anticoagulants and therefore most of these patients would actually fit the criteria for acceptable SUP use. And these patients were not associated with significant SUP misuse (Issa et al., 2012), which is consistent with our study.

Our study shows that the concurrent use of corticosteroids or anticoagulants is a predictor of underprescription of SUP. Prior studies have demonstrated PPIs underprescription and overprescription to be positively and negatively associated with systemic corticosteroids, respectively (Schepisi et al., 2016), which is consistent with our study. A study had shown that inappropriate SUP increased twofold in patients concomitantly using corticosteroids or anticoagulants (Issa et al., 2012). Furthermore, the concomitant use of anticoagulants was also a significant independent predictor of guideline-noncompliance prescribing of PPIs in another study (Eid et al., 2010).

Our results suggest that unemployed inpatients are more likely to be under-prescribed ASMs for SUP, while unemployed and retired inpatients are less likely to be overprescribed ASMs for SUP. The most likely reason for this is that economic characteristics among different populations are factors which might influence clinicians’ prescribing behavior in underdeveloped regions where this study has been conducted. Because unemployed and retired inpatients tended to have lower incomes and more barriers to access affordability of drugs than employed inpatients, it is possible that the prescribers were aware of the economic situation of such inpatients and generally avoided prescribing medications for these patients. But there is little information indicative of any association of SUP medication with employment status in the available literature. The relationship between employment status and inappropriate prescription of SUP needs further research to build upon our findings in the future.

Prolonged hospitalization was found to be predictive of increased likelihood of inappropriate prescription of SUP in inpatients in our study. One study had noted that the duration of hospital stay was a significant factor for AST misuse (Issa et al., 2012). But literature has also suggested that the proportion of correct use of ASMs has increased, while the proportion of misuse has decreased with prolonged hospitalization (Mayet, 2007), which is contrary to the results obtained in our study.

The reasons why clinicians prescribed SUP inappropriately were multifactorial. First, the fear of development of stress ulcer syndrome in non-ICU patients who were not on SUP therapy might be largely unreasonable, as the overall incidence of bleeding events seemed low (Allen et al., 2004; Hussain et al., 2010). Due to the tense relationship between doctors and patients in China, doctors had to prescribe SUP therapy for low-risk inpatients so as to protect themselves from litigation (Luo et al., 2018). The incidence of an adverse reaction related to ASMs has not been high, and for this reason, doctors have believed PPIs to be safe (Hussain et al., 2010). The incidence of serious clinical adverse reactions in adults has been low when PPIs and H_2_RAs were used for a short time (ASHP Therapeutic Guidelines on Stress Ulcer Prophylaxis, 1999). However, physicians must take into account the possible risk of the side effect when prescribing PPIs. Last but not the least, doctors might not have prescription awareness of the existing guidelines, and it is conceivable that SUP has been routinely prescribed to their patients (Heidelbaugh and Inadomi, 2006; Voukelatou et al., 2019).

### Strengths and Limitations

To the best of our knowledge, current studies in China mainly focus on the irrational use of PPIs, and this is the first study to identify the associated factors of inappropriate SUP medication for inpatients of fracture who have underwent surgical operations in the orthopedics department. We believe that this study will help the researchers and policymakers understand the prescription behavior of clinicians comprehensively and provide effective measures for SUP management. Our study has several limitations. First, as only a single tertiary hospital was surveyed, it might create a bias due to the small size of inpatient sampling. However, we believe the findings of our study are worthy of reference for other hospitals in China. The second limitation is that differences in the incidence of SU between fracture operations of inpatients might lead to bias. This study has focused on inpatients of fracture who underwent surgical operations outside the ICU ward, and inpatients were not stratified according to the types of fracture operations, which needs to be further evaluated in future studies. Furthermore, the clinical practice guidelines for the evaluation of appropriate SUP medication in surgery patients has not been established as consensus statements. Therefore, we have established the evaluation criteria according to evidence-based recommendations.

### Practical Implications

We should pay more attention to the prevalence of inappropriate prescribing patterns of SUP. The presence of patient risk factors for stress ulcer syndrome should determine the need for SUP. Institution-specific recommendations must be formulated to help clinicians identify appropriate candidates for SUP medication (Allen et al., 2004; Hussain et al., 2010). Continuous education programs for clinicians detailing evidence-based indications for SUP and the adverse reaction of AST are required to correct doctors’ misunderstandings (Hussain et al., 2010; Savarino et al., 2018). The intervention of clinical pharmacists could decrease the inappropriate usage of ASMs, as well as drug expenditures and the risk of adverse events, effectively (Hussain et al., 2010; Jarchow-Macdonald and Mangoni, 2013; Buckley et al., 2015; Masood et al., 2018; Tandun et al., 2019). Clinical pharmacists could help strengthen regulation of clinical application of PPIs. For future studies, more comprehensive information on the irrational use of PPIs should be collected and drug-related problems (DRPs) should be investigated, so as to provide a reference for PPIs stewardship.

## Conclusion

This cross-sectional observational study has confirmed that approximately 33.1% of the 1,200 inpatients of fracture who underwent surgical operations in the orthopedics department might not require intravenous PPIs for SUP on a routine basis. Additionally, the prevalence of inappropriately prescribed PPIs for SUP had increased unnecessary costs and the potential risk of adverse events. Furthermore, we delineated the associated factors with inappropriate SUP medications, which indicates the need for more information for clinicians on rationality and efficiency of their prescribing practices. Age above 65 years and prolonged hospitalization were associated with overprescription of SUP. Conversely, prolonged hospitalization, the concurrent use of systemic corticosteroids or anticoagulants, and unemployed status in inpatients were positively associated with underprescription of SUP. Effective intervention strategies should be executed by clinical pharmacists to reduce improper SUP medication and attain substantial cost savings without impairment of patient outcome.

## Data Availability

The original contributions presented in the study are included in the article/Supplementary Material, further inquiries can be directed to the corresponding author.

## Abbreviations

SUP, stress ulcer prophylaxis; SRMD, stress-related mucosal disease; PPIs, proton pump inhibitors; AST, acid suppression therapy; SU, stress ulceration; ICU, intensive care unit; ASMs, acid suppressive medications; NSAIDs, non-steroidal anti-inflammatory drugs; DDD, defined daily dose; DDDs/100 PDs, defined daily doses per 100 patient-days; H_2_RAs, histamine-2-receptor antagonists
