# Supplementary material for: Appropriateness and Associated Factors of Stress Ulcer Prophylaxis for Surgical Inpatients of Orthopedics Department in a Tertiary Hospital: A Cross-Sectional Study
Source: Front Pharmacol. 2022 Jun 2;13:881063. doi: 10.3389/fphar.2022.881063 (PMC9203048; doi:10.3389/fphar.2022.881063)
Supplement: Supplementary file 1 [file Table1.DOCX]

**TABLE S1** The diagnosis of inpatients with acid suppressive medications for SUP (n=893)

| **Diagnosis** | **Number of cases**  **n (%)** | **Number of cases received surgical operation more than 3 hours**  **n (%)** |
| --- | --- | --- |
| Fracture of the femoral neck | 102(11.4) | 32(31.4) |
| Intertrochanteric fracture of femur | 98(11.0) | 10(10.2) |
| Fracture of the ankle | 84(9.4) | 9(10.7) |
| Fracture of the shaft of the humerus | 76(8.5) | 27(35.5) |
| Fracture of the spine | 74(8.3) | 12(16.2) |
| Fracture of the tibia and fibula | 68(7.6) | 26(38.2) |
| The proximal and distal femoral fractures | 66(7.4) | 24(36.4) |
| Fracture of the patella | 56(6.3) | 9(16.1) |
| Fracture of the pelvis | 55(6.2) | 11(20.0) |
| Fracture of tibial plateau | 40(4.5) | 9(22.5) |
| Fracture of the shaft of the femur | 28(3.1) | 6(21.4) |
| Fracture of the clavicle | 28(3.1) | 3(10.7) |
| Fracture of the calcaneum | 27(3.0) | 7(25.9) |
| Fracture of the radius and ulna | 21(2.4) | 5(23.8) |
| Fracture of the distal radius | 21(2.4) | 1(4.8) |
| Fracture of the acetabulum | 18(2.0) | 7(38.9) |
| Fracture of the metatarsal | 10(1.1) | 0(0) |
| Fracture of metacarpal bones | 9(1.0) | 1(11.1) |
| Fracture of the phalanx | 3(0.3) | 1(33.3) |
| Others | 9(1.0) | 1(11.1) |
| Overall | 893(100.0) | 201(22.5) |
