# Supplementary material for: Appropriateness and Associated Factors of Stress Ulcer Prophylaxis for Surgical Inpatients of Orthopedics Department in a Tertiary Hospital: A Cross-Sectional Study
Source: Front Pharmacol. 2022 Jun 2;13:881063. doi: 10.3389/fphar.2022.881063 (PMC9203048; doi:10.3389/fphar.2022.881063)
Supplement: Supplementary file 2 [file Table2.DOCX]

**TABLE S2** The diagnosis of inpatients without acid suppressive medications for SUP (n=307)

| **Diagnosis** | **Number of cases**  **n (%)** | **Number of cases received surgical operation more than 3 hours**  **n (%)** |
| --- | --- | --- |
| Fracture of the ankle | 42(13.7) | 11(26.2) |
| Fracture of the patella | 39(12.7) | 6(15.4) |
| Fracture of the radius and ulna | 33(10.7) | 3(9.1) |
| Fracture of the tibia and fibula | 31(10.1) | 12(38.7) |
| Fracture of the shaft of the humerus | 29(9.4) | 12(41.4) |
| Fracture of the shaft of the femur | 25(8.1) | 5(20.0) |
| Fracture of the clavicle | 25(8.1) | 0(0) |
| Fracture of the spine | 18(5.9) | 6(33.3) |
| Fracture of metacarpal bones | 14(4.6) | 0(0) |
| Fracture of the metatarsal | 12(3.9) | 2(16.7) |
| Fracture of tibial plateau | 11(3.6) | 3(27.3) |
| Fracture of the femoral neck | 9(2.9) | 5(55.6) |
| Fracture of the pelvis | 7(2.3) | 2(28.6) |
| Fracture of the calcaneum | 5(1.6) | 0(0) |
| Fracture of the acetabulum | 2(0.7) | 2(100.0) |
| Others | 5(1.6) | 1(20.0) |
| Overall | 307(100.0) | 70(22.8) |
